# Efficacy and executive function of solution-focused brief therapy on adolescent depression

**DOI:** 10.3389/fpsyt.2024.1246986

**Published:** 2024-03-08

**Authors:** Haisi Chen, Mengmeng Zhou, Li Han, Advaith Manoharasetty, Zhenghe Yu, Hong Luo

**Affiliations:** ^1^Affiliated Mental Health Center & Hangzhou Seventh People’s Hospital, Zhejiang University School of Medicine, Hangzhou, China; ^2^Internal Medicine Department, Hangzhou Linping District Maternal and Child Care Hospital, Hangzhou, China; ^3^Institute for International Education, Zhejiang Chinese Medical University, Hangzhou, China

**Keywords:** Solution-Focused Brief therapy, psychodynamic psychotherapy, adolescent, Major depressive disorder, Functional near-infrared spectroscopy

## Abstract

**Objective:**

To investigate the efficacy and impact on executive function of Solution-Focused Brief Therapy (SFBT) in treating Major Depressive Disorder (MDD) in adolescents.

**Methods:**

A total of 129 adolescents diagnosed with MDD were enrolled in the study. Out of these, 28 adolescents were assigned to the SFBT group, while 25 were part of the Active Control group (AC group), receiving psychodynamic psychotherapy. Executive function, depressive and anxiety symptoms were assessed at baseline, at the time of the third intervention, the sixth intervention, and the 10th intervention.

**Results:**

After the third intervention, the scores of the Patient Health Questionnaire-9 (PHQ-9) and Generalized Anxiety Disorder-7 (GAD-7) of the participants in the SFBT group decreased significantly, which had the cumulative effect at the 6th and 10th interventions. The verbal fluency task (VFT) performances of the SFBT group participants yielded significantly higher scores after the third intervention and remained increasing at the 6th and 10th interventions. The AC group steadily decreased after the intervention. Analysis of functional near-infrared spectroscopy (fNIRS) data revealed a progressive and significant increase in the average oxyhemoglobin (oxy-Hb) levels in the dorsolateral prefrontal cortex (DLPFC) in the SFBT group compared to the AC group after the 10th intervention.

**Conclusions:**

SFBT might improve depressive and anxiety symptoms as well as executive function of adolescent depression.

**Clinical trial registration:**

https://www.chictr.org.cn, identifier ChiCTR2300067909.

## Introduction

According to the study, many adults with depression recall that their first depressive episode dates back to their teenage years (1). The diagnosis and treatment of Major Depressive Disorder (MDD) in adolescents are complicated by factors such as diminished self-awareness of depressive symptoms, heightened emotional volatility, and the subjective nature of clinical evaluations. Although antidepressants are the first-line treatment for depression, 50% of patients do not respond to early treatment, and it is difficult to predict the response to treatment at the onset (2). The prevalence of adolescent depression is 5.2~7.5%, and with higher prevalence among females than males (3). The latest survey report on the prevalence of mental illness in children and adolescents in China shows that the prevalence of depression in adolescents is as high as 3% (4). Numerous prevalence studies suggest that 8 to 20% adolescent depression is associated with poor relationships, academic failure, substance abuse, major depressive disorder, suicide, and other psychiatric disorders in adulthood, which predict an increased risk of lifelong dysfunction (5). However, current treatment options for adolescents suffered from depression remain limited.

According to the recommendations, psychotherapy is considered the first choice of initial treatment for adolescents with mild to moderate depression (6). Given guidelines recommend a combination of antidepressants and a recognized psychotherapy for moderate-to-severe adolescent depression. Most studies focused on Cognitive Behavioral Therapy or Interpersonal Therapy, but none of the studies have focused on Solution-Focused Brief Therapy (SFBT) as the treatment of adolescent depression (7). SFBT shifts the focus away from problem formation and problem resolution to participants’ future goals, strengths, and resiliency. In SFBT, a professional collaborates with the client to look for solutions to obtain goals and strongly stresses the client’s autonomy and competencies to achieve them (8). According to several meta-analyses and reviews, it has positive effects in a broad range of settings and problem areas (8–10). The latest and most extensive analysis, which includes five studies, centers on adult depression as a result (8). However, none of these investigations addressed depression in adolescents.

Functional near-infrared spectroscopy (fNIRS) is a real-time and non-invasive neuroimaging technique. Many studies have combined fNIRS and the verbal fluency task (VFT) to evaluate executive function, which refers to a series of neuropsychological abilities including maintaining attention, and flexibly changing goals and plans according to changing events (11). The prefrontal cortex is a key center for combining cognitive control and emotion regulation, which is closely associated with cognitive function and emotion regulation (12). Findings from several prior research efforts have indicated that patients with MDD exhibit reduced prefrontal cortex oxyhemoglobin (oxy-Hb) responses and suboptimal activation levels during cognitive challenges (13).

The present study was designed to remedy the relative lack of attention paid to the efficacy and executive function of SFBT on emotional regulation in adolescent depression. It was a preliminary exploration of efficacy and executive function of SFBT in clinical practice from the aspects of subjective scale evaluation and objective evaluation via near-infrared imaging spectrum.

## Method

### Participants

Consecutive outpatients with MDD were enrolled (N=129) from November 2021, underwent screening over a period of 12 months. To be eligible for this study, participants had to satisfy following inclusionary criteria (1): patients were aged 12–18 years (2); patients with DSM-IV clinical criteria for the major depressive episode using the Mini-International Neuropsychiatric Interview (MINI) by two psychiatrists who had at least 5 years of clinical experience. Important exclusion criteria were as follows (1): patients were diagnosed with mental retardation, psychosis, bipolar disorder, or obsessive–compulsive disorder, post traumatic stress disorder or eating disorders defined by DSM-IV; (2) The treatment history of electroconvulsive therapy or psychotherapy; (3) The presence of self-injury behavior or suicidal attempts during interview.

This study was registered at https://www.chictr.org.cn and the registration identification number was ChiCTR2300067909. The study was approved by the Institutional Review Board (IRB) of the Affiliated Hospital of Hangzhou Normal University (Hangzhou, China; 2022 (E2)-HS-076).

### Treatments

#### The therapies

The study was non-randomized concurrent controlled trial (Non-R), for the consideration of various factors such as the patient’s condition, autonomous selectivity and flexible treatment time. Participants were allocated to either the SFBT group or the Active Control group (AC group), which received psychodynamic psychotherapy, according to their preference for the therapeutic approach. However, participants were kept unaware of the specific details of the psychological treatment they received, yet informed about the study’s general purpose and potential implications. For the SFBT group, subjects received solution-focused therapy a brief resource-oriented and goal-focused therapeutic approach, which helps clients change by constructing solutions (14). The technique includes the search for pre-session change, miracle and scaling questions, exploration of exceptions, use of a one-way mirror and consulting break, positive feedback and home assignments. The orientation was based on an approach developed by De Shazer and Berg (15).

For the AC group, subjects received short-term psychodynamic psychotherapy, a brief, focal, transference-based therapeutic approach which helps patients by exploring and working through specific intra-psychic and interpersonal conflicts. Short-term psychodynamic psychotherapy is characterized by the exploration of a focus, which can be identified by both the therapist and the patient (16).

Ensure that all participants follow the same procedures and receive the same instructions, conducted at a separate physical location from the treatment sessions, regardless of their group assignment. The SFBT group and AC group received different intervention modalities, and the participants in the two groups remained consistent in the duration of a single intervention (40-60 minutes/session). But the interval between each intervention was not fixed, for it was decided by the therapist and patient discussion.

#### The therapists

The therapist in SFBT group had been trained for the method and received a qualification in solution-focused therapy provided by a local institute and had nearly 20 years of experience in solution-focused therapy without any training in psychodynamic psychotherapy. The therapist providing psychodynamic psychotherapy had received standard training in psychoanalytically orientated psychotherapy that was approved by one of the psychoanalytic or psychodynamic training institutes without any experience of solution-focused therapy.

In psychodynamic psychotherapies, the therapies were conducted in accordance with clinical practice, where the therapists might modify their interventions according to a patient’s needs within the framework of psychodynamic therapies. Accordingly, no adherence monitoring was organized and no manuals were used.

### Measures

#### Clinical measurement

The measurements were carried out as ratings based on interviews and mental health questionnaires. such as the Patient Health Questionnaire-9 (PHQ-9) and Generalized Anxiety Disorder-7 (GAD-7). The assessments were completed at baseline examination and during follow-up at 3rd, 6th, 10th intervention.

The PHQ-9 (17) is a nine-item instrument that screens for the presence and severity of depression. It asks the patient about their experiences over the preceding 2 weeks. Scores range from 0 to 27. In general, a score ≥10 suggests depression.

GAD-7 (18) is a self-report instrument to assess the severity of anxiety in general. GAD-7 has seven items and scores range from 0 to 21. Greater scores indicate greater anxiety over the preceding 2 weeks.

The verbal fluency task (VFT) has been widely used to study executive capability in depression, which has been purported to be associated with cognitive impairment (19, 20). During the pre- and post-task baseline periods, the participants were asked to repeat counting from 1 to 5. During the task period, the participants were required to construct as many phrases as possible using three commonly used characters, such as “蓝” (blue), “大” (big), and “天” (sky). The three given characters changed every 20 s during the task period to reduce the time during which the subjects were silent (21).

#### NIRS measurement

The participants were seated comfortably in a quiet room, and hemoglobin concentrations were measured using a multi-channel near infrared optical imaging system (NirScan, Danyang Huichuang Medical Equipment Co., Ltd., China). The FPz channel was used (10/20 international system) as the center of the middle probe; a total of 19 single-distance (SD) probes (consisting of 7 sources and 7 detectors) with a fixed 3-cm inter-probe distance were placed to cover each subject’s bilateral prefrontal cortex. The integral value of oxy-Hb of the bilateral frontal lobe were used to characterize brain activation in adolescent depression.

### Data processing and analysis

We used the NirSpark software package (HuiChuang, China) to analyze NIRS data. Data were preprocessed via the following steps. Motion artifacts were corrected by a moving SD and a cubic spline interpolation method. A bandpass filter with cut-off frequencies of 0.01–0.20 Hz was used to remove physiological noise (e.g., respiration, cardiac activity, and low-frequency signal drift). The modified Beer-Lambert law was used to convert optical densities into changes in oxy-Hb and deoxy-Hb concentrations. Oxy-Hb was used as the primary indicator in this analysis because it generally has a better signal-to-noise ratio than Deoxy-Hb (22). We used a 60-s task period of constructing phrases as the time window to analyze mean oxy-Hb changes. Linear fitting was applied to the data between these two baselines. The primary outcomes measured were the levels of depression and anxiety, alongside the average active oxy-Hb values of fNIRs during the VFT. The severity of depression was quantified by the PHQ-9 and anxiety symptoms were assessed by the GAD-7.

### Statistical analysis

Statistical analyses were performed using SPSS Version 25.0. The NirSpark software package, GraphPad Prism 8, and Adobe Illustrator software were used to generate figures and graphs. Independent sample t-tests were used to investigate the differences between groups. A paired sample t-test or repeated-measures analysis of variance (ANOVA) was performed for standard distribution data. Data normality was tested via the Shapiro-Wilk test. The demographic and clinical data were compared by t-tests or chi-square tests. Student’s t-test was used to analyze differences in age and education level between the SFBT group and the AC group at baseline.

We used a two-way mixed ANOVA with different groups (SFBT group *vs*. AC group) as the between-subject factor and time (T0、T3、T6、T10) as the within-subject factor to analyze the effect of the SFBT therapy intervention on the oxy-Hb values. The two-way mixed ANOVA included a 4 (time point: T0、T3、T6、T10) × 2 (group: SFBT group and AC group) design.

Functional connectivity was analyzed by performing Spearman’s correlation between the time series of each channel-to-channel pair. A p-value less than 0.05 was considered statistically significant, and all p-values were two-tailed.

## Results

**Figure 1:**
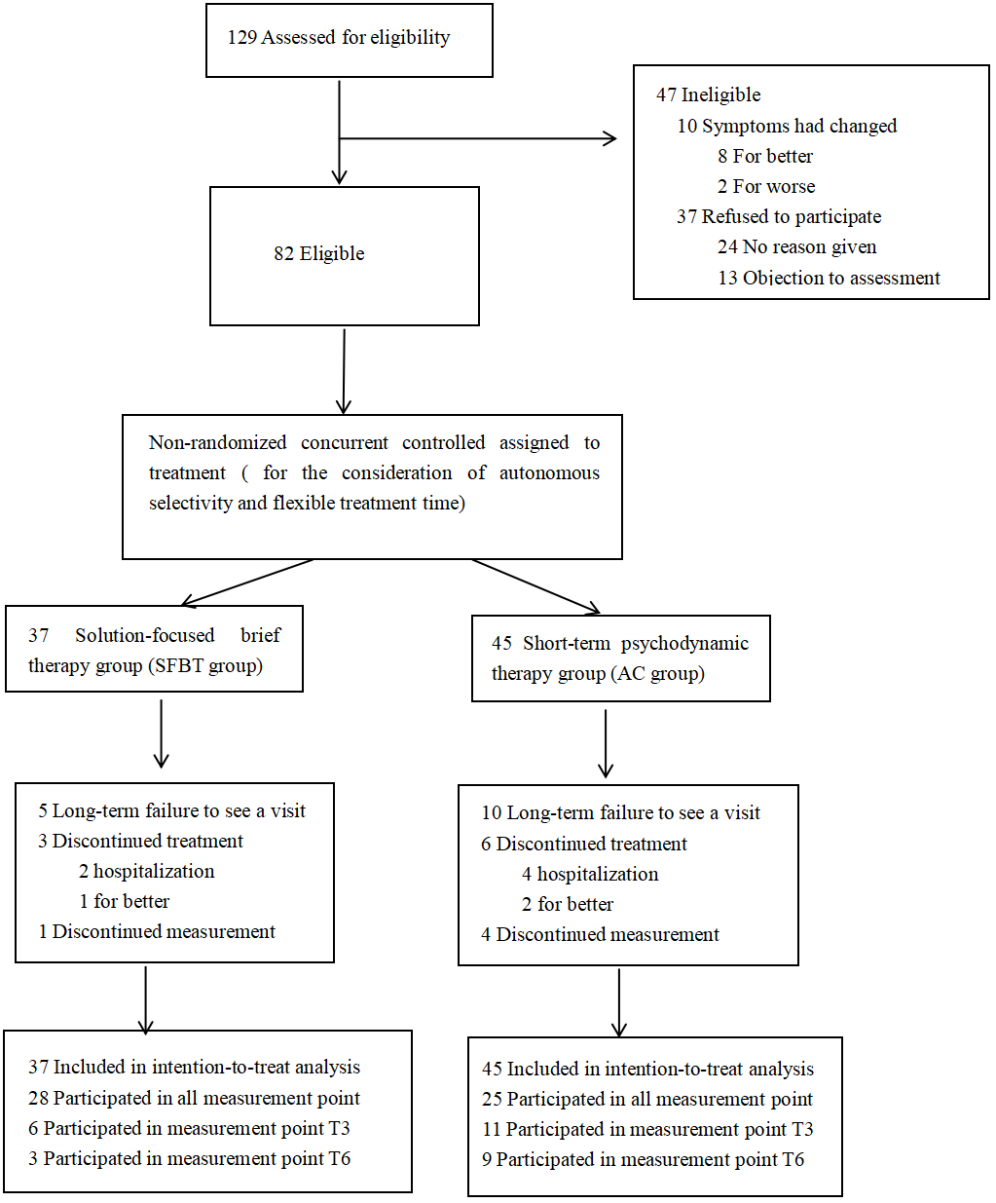
Number of patients assessed for eligibility, assigned to study group, and who completed the protocol.

A total of 129 adolescent depression who met the inclusion criteria were enrolled and underwent baseline measurements. 47 patients were excluded for the following reasons (1): they discontinued treatment for getting recovered (N=8); (2) they had to be hospitalized for suicidal attempt (N=2); (3) they refused to participate in the study (n = 37) (shown in **Figure 1**). Of the patients starting the therapy a total of 29 patients discontinued the treatment prematurely.

The remaining 82 participants underwent long-term follow-up. Among the 37 participants who enrolled in the SFBT group, 28 of them completed the full 10-session intervention program and provided comprehensive post-test data at T3, T6, and T10. In the AC group, which initially had 45 participants, 25 persisted through to the final intervention and also provided complete post-test data at the same three time points. As shown in **Figure 1**.

### Demographic and clinical characteristics

The demographic characteristics of participants in each group are presented in **Table 1**. The SFBT group included 9 males and 19 females (mean age = 15.11 years; SD = 1.42). The AC group included 5 males and 20 females (mean age = 14.80 years; SD = 1.41). Both groups showed no difference in age, gender or education (*P* > 0.05). No statistically significant differences among treatment groups were found with respect to medication at baseline. As shown in **Table 1**.

**Table 1:** Demographic data of the SFBT group and AC group (mean ± SD).

	SFBT group	AC group	*Z/χ^2^*	*P*
	(n=28)	(n=25)		
Demographics				
Gender (female/male)	9/19	5/20	1.002	0.317^a^
Age (years)	15.11 ± 1.42	14.80 ± 1.41	-0.839	0.401^b^
Education (years)	9.64 ± 1.45	8.76 ± 2.13	-1.555	0.120^b^
Medication				
Antidepressant	28/0	25/0	/	/
Antipsychotic	10/18	10/15	0.103	0.748^a^
Benzodiazepine	8/20	4/21	1.192	0.275^a^

Values are mean ± SD unless otherwise stated. ^a^Pearson χ^2^ test; ^b^ Independent sample t-test.

### Clinical data and VFT task performance of participants before and after therapy

#### Clinical data

The results showed that the main effect of time point was significant [*F* (3,49)=9.865, *P*<0.001,*η*^2 ^= 0.377], meaning that PHQ-9 scores decreased after therapy in all participants (see **Table 2**). The main effect of Group was not significant [*F* (1, 51)=2.293, *P*=0.136]. The interaction between Group and Time point was significant [*F* (3, 49)=2.969, *P*=0.056, *η*^2 ^= 0.142]. Simple effect analysis revealed that the PHQ-9 scores in both two groups were significantly reduced at T3, T6, T10 assessment compared with the T0 point (*P* < 0.05). The simple effect analysis also showed no significant difference in PHQ-9 scores between the two groups at T0, T3, and T10. However, at T6, the SFBT group had significantly lower PHQ-9 scores than the AC group (*P*=0.006).

**Table 2:** Clinical data of SFBT group and controls (mean ± SD).

Condition	Time point	SFBT(n=28)	AC(n=25)
PHQ-9	T0	18.32 ± 5.40	17.80 ± 5.15
	T3	15.43 ± 5.71^b^	17.12 ± 6.33
	T6	13.29 ± 5.51^ce^	17.80 ± 6.03
	T10	12.11 ± 6.25^c^	14.32 ± 7.16^b^
GAD-7	T0	13.54 ± 4.42	14.52 ± 5.14
	T3	11.14 ± 5.05^c^	12.76 ± 5.44^c^
	T6	11.04 ± 4.75^b^	13.88 ± 4.52^b^
	T10	9.54 ± 5.33^c^	11.28 ± 6.07^c^

SFBT, Solution-focused brief therapy; AC, Psychodynamic therapy; PHQ-9, the Patient Health Questionnaire; GAD-7, Generalized Anxiety Disorder-7.

Values are mean ± SD; with repeated-measures analysis of variance. 0.05<^a^P<0.1, ^b^P<0.05, ^c^P<0.001 relative to baseline;0.05<^d^P<0.1, ^e^P<0.05, ^f^P<0.001 between groups.

The GAD-7 was employed to assess the severity of anxiety in the SFBT group and AC group. The results showed that the main effect of time point was significant [*F* (3,49)=11.148, *P*<0.001,*η*^2 = ^0.406], meaning that GAD-7 scores decreased after therapy in all participants (refer to **Table 2**). The main effect of Group was not significant [*F* (1, 51)=2.618, *P*=0.112]. The interaction between Group and Time point was not significant [*F* (3, 49)=0.589, *P*=0.625].

Additional *post hoc* analyses found that the GAD-7 scores of T3, T6 and T10 were significantly lower than that of T0 (*P<*0.05). The GAD-7 scores of T10 showed a marginal significant lower than the score at T3 (*P*=0.061), and was significantly lower than the scores at T6 (*P*=0.002). No significant differences were found in the GAD-7 scores between the other time points (*P*>0.1). As shown in **Table 2**.

#### VFT task performance

During the VFT task, the number of words generated by the SFBT and AC groups are summarized in **Table 3**. The results showed that the main effect of Group was significant [*F* (1,51) = 8.752, *P* = 0.005, *η*^2 ^= 0.146], meaning that the performances on the VFT task in the SFBT group were higher than those in the AC group. The main effect of Time point was not significant [*F* (3,49) = 0.143, *P* = 0.934]. The interaction between Group and Time point was significant [*F* (3,49) = 4.473, *P* = 0.005, *η*^2 ^= 0.081]. There was no significant difference in the performances on the VFT task at T0 (*P*=0.980), while the SFBT group were significantly higher than those in the AC group in the time of T3, T6 and T10 (*P*<0.05). As shown in **Table 3**.

**Table 3:** VFT task performance and the mean integral value of oxy-Hb of the frontal lobe area during VFT (mean ± SD).

Condition	Time point	SFBT(n=28)	AC(n=25)
VFT	T0	9.46 ± 3.64	9.44 ± 3.40
	T3	11.18 ± 3.85^be^	8.48 ± 4.85
	T6	11.21 ± 4.38^be^	8.08 ± 3.40
	T10	11.68 ± 4.75^be^	7.64 ± 3.07^b^
Integral value	T0	39.23 ± 69.16	14.91 ± 61.20
	T3	-23.96 ± 70.59^cd^	15.52 ± 78.81
	T6	86.05 ± 67.82^bf^	9.37 ± 55.45
	T10	153.53 ± 77.99^cf^	22.80 ± 58.25

Values are mean ± SD; with repeated-measures analysis of variance. 0.05<^a^P<0.1, ^b^P<0.05, ^c^P<0.001 relative to baseline;0.05<^d^P<0.1, ^e^P<0.05, ^f^P<0.001 between groups.

#### The integral value of oxy-Hb in the frontal lobe

The results showed that the main effect of Time point was significant [*F* (1,51)=12.559, *P*=0.001,*η*^2 ^= 0.198] and the interaction between Group and Time point was significant [*F* (3, 49)=1.960, *P*<0.001, *η*^2 ^= 0.494]. The integral value of oxy-Hb at T10 was significantly higher than that at T0, T3, T6 (*P*<0.05) in SFBT group. While there was no significant difference in the integral value of oxy-Hb in AC group at the time point of T0, T3, T6 and T10 (*P*>0.1). As shown in **Table 3**.

**Figure 2:**
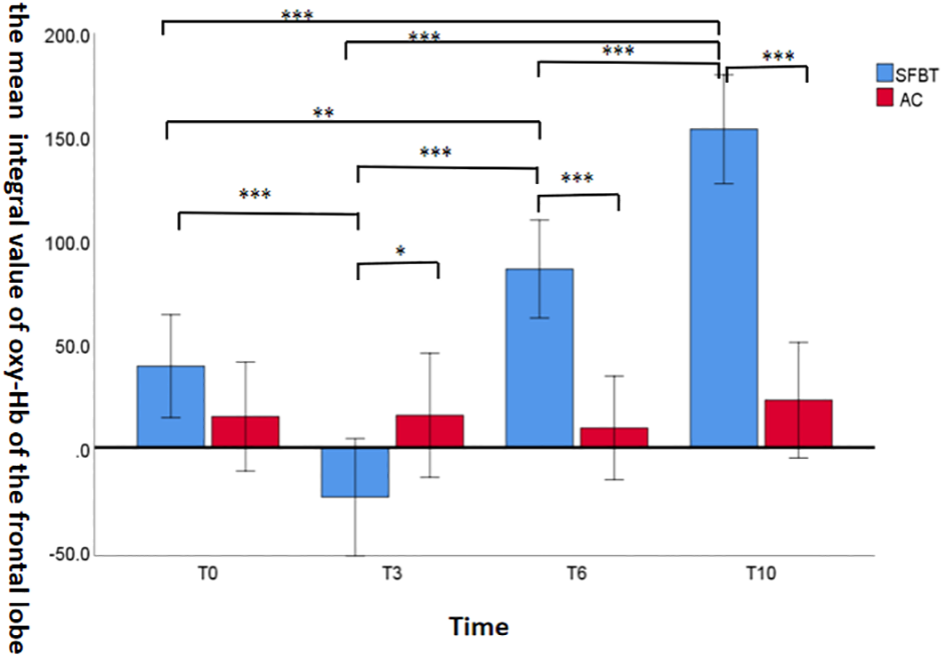
The mean integral value of oxy-Hb of the frontal lobe area during the performance of the task of VFT across the groups. (*P<0.1, **P<0.05, ***P<0.001).

**Figure 2** demonstrates the integral value of oxy-Hb in the frontal lobe of the SFBT group and AC group during the task of VFT. It can be seen that there was no significant difference in the integral value of oxy-Hb between the SFBT group and the AC group at baseline. However, the SFBT group exhibited a marginally significant lower integral value of oxy-Hb compared to the AC group (*P*=0.060). At T6 and T10, the integral value of oxy-Hb in the SFBT group was significantly higher than that in the AC group (*P*<0.001).

#### The main effect of time point of fNIRS data analysis

The results showed that the main effect of Time point was significant in channel 17 [*F* (3,49) = 3.687, *P* = 0.013, *η*^2 ^= 0.067]. Additional *post hoc* analyses found that the mean active Oxy-Hb values at T10 was significantly higher than that at T3 (*P*=0.004), and was marginal significantly higher than those at T0 and T6 (*P*=0.056, *P*=0.062). As shown in **Table 4**.

**Table 4:** The mean oxy-Hb changes in the five channels across the groups.(mean ± SD).

	SFBT(n=28)	AC(n=25)
CH1 oxy-Hb		
T0	-0.066893 ± 0.421249	-0.146590 ± 0.553377
T3	-0.092196 ± 0.611896^d^	-0.258232 ± 0.289063
T6	-0.070937 ± 0.363762^d^	-0.157184 ± 0.412706
T10	-0.084603 ± 0.379781^d^	-0.258250 ± 0.382398
CH3 oxy-Hb		
T0	-0.040874 ± 0.310211	-0.109224 ± 0.583864
T3	0.177522 ± 0.601971^e^	-0.161760 ± 0.343596
T6	0.038321 ± 0.533234^e^	-0.081278 ± 0.554993
T10	-0.035575 ± 0.403430^e^	-0.324650 ± 0.470708
CH11 oxy-Hb		
T0	-0.096095 ± 0.437341	-0.180457 ± 0.310932
T3	0.049087 ± 0.604841^e^	-0.154253 ± 0.364345
T6	-0.054372 ± 0.595310^e^	-0.084196 ± 0.417738
T10	0.104943 ± 0.473885^e^	-0.306312 ± 0.468670
CH12 oxy-Hb		
T0	-0.235744 ± 0.919030	-0.092010 ± 0.203480
T3	-0.076049 ± 0.390389	-0.205110 ± 0.258400
T6	-0.172352 ± 0.482569	-0.047436 ± 0.470329
T10	0.005516 ± 0.434714^e^	-0.244675 ± 0.400495
CH17 oxy-Hb		
T0	-0.034038 ± 0.248337	-0.212178 ± 0.633903
T3	-0.169161 ± 0.394913	-0.256328 ± 0.497363
T6	-0.091867 ± 0.364183	-0.088492 ± 0.399559
T10	0.038872 ± 0.365417^a^	0.141596 ± 0.776911^a^

Values are mean ± SD; with repeated-measures analysis of variance. 0.05<^a^P<0.1, ^b^P<0.05, ^c^P<0.001 relative to baseline;0.05<^d^P<0.1, ^e^P<0.05, ^f^P<0.001 between groups.

#### The main effect of group

The results showed that the main effect of Group was significant in channel 3 [*F* (1,51) = 8.116, *P* = 0.006, *η*^2 ^= 0.137], channel 11 [*F* (1,51) = 6.849, *P* = 0.012, *η*^2 ^= 0.118], indicating that the mean active oxy-Hb values in the SFBT group were higher than those in the AC group. Channel 1 showed a marginal significant change [*F* (1,51) = 3.705, *P* = 0.060, *η*^2 ^= 0.068].

#### The interaction between group and time point

**Figure 3:**
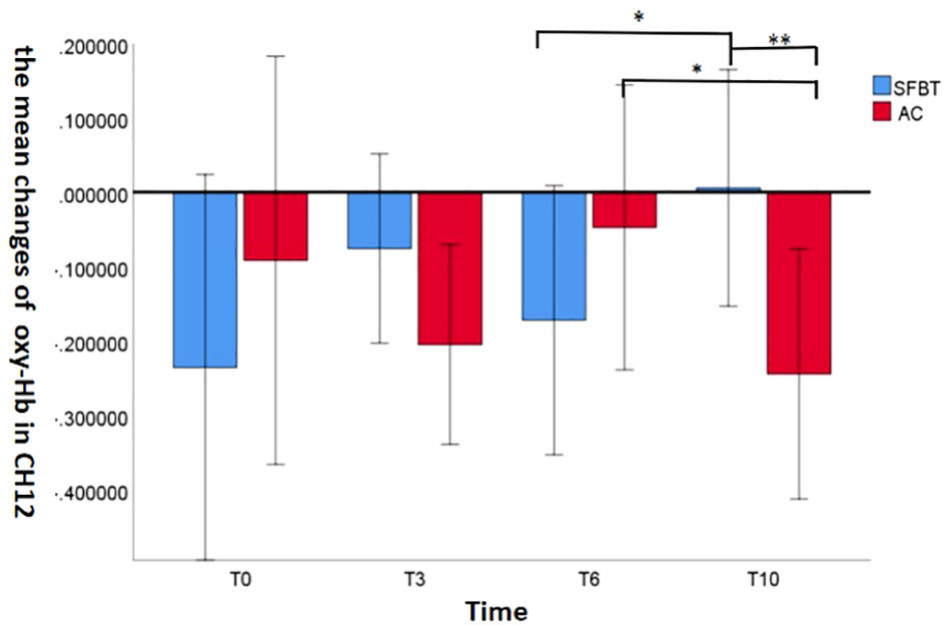
The mean Oxy-Hb changes in the channel 12 across the groups. (*P<0.1, **P<0.05, ***P<0.001).

The interaction between Group and Time point showed that Channel 12 showed a marginal significant change [*F* (3,49) = 2.459, *P* = 0.074, *η*^2 ^= 0.131]. The mean active oxy-Hb values in participants are shown in **Figure 3**.

Simple effect analyses revealed that the mean active oxy-Hb value in channel 12 in SFBT group was significantly increased at the T10 assessment compared with the T6 (*P* = 0.082). While the mean active oxy-Hb value in channel 12 in the AC group was significant decreased at the T10 assessment compared with the T6 (*P* = 0.069). According to the simple effect analysis of treatment time, the results demonstrated that the mean active oxy-Hb value in the SFBT group was significantly higher than that in the AC group at the T10 (*P* = 0.035), but no significant change was found at the T0, T3, T6 between the groups (*P*>0.1).

## Discussion

Our finding showed that SFBT produced a faster symptom reduction than psychodynamic therapy during the entire 10 times of treatment and has not been reported previously. The score of PHQ-9 in the SFBT group significantly decreased at T3 compared to the baseline and showed a stable decline pattern at T6 and T10. Meanwhile the score of PHQ-9 in the AC group decreased only after the 10th intervention and no significant difference was found at T3 and T6. The results confirmed the clinical expectation of greater beneficial effects from short-term SFBT treatment. A quasi-experimental study undertaken to explore the effectiveness of psychoanalysis, long-term and short-term psychotherapy during a 5-year follow-up supported that short-term therapies as SFBT were more effective than psychoanalysis during the first year, whereas the long-term therapy was more effective after 3 years of follow-up (23). In addition, a pilot study revealed that the solution-focused questions in SFBT were overall more effective, providing the same benefits as the problem-focused condition while also increasing positive affect and participants’ understanding of the nature of the problem, suggesting that coaches aim for solution-focused theme in work to build self-efficacy (24).

To the best of our knowledge, this study is the first to use fNIRS to investigate the efficacy and executive function of SFBT on oxy-Hb changes in adolescent depression. Our results showed that the mean active oxy-Hb values of channels 3, 11 and 12 (mainly consisted of dorsolateral prefrontal cortical regions) gradually and significantly increased in the SFBT group compared to the AC group after 10th therapy sessions. These results indicated that SFBT is more effective in improving brain function in adolescent depression than psychodynamic therapy within a brief period. The most relevant cortex is the dorsolateral prefrontal cortex (DLPFC). Recent neuroimaging studies on adolescent depression offered evidence of specific functional abnormalities in the DLPFC. Xie et al. demonstrated that the neuropsychopathological factor mainly encompassed prefrontal cortical circuits, such as the superior medial frontal, salience and frontoparietal networks, and delayed development of the prefrontal cortex that further led to poor executive function (25). Some researchers found that adolescent depression had hypofunction in the DLPFC and the frontotemporal cortex during the VFT, which made it difficult for the patients to make plans and decisions and to susceptible to affective disorders (26–29). Consistent to previous findings, researchers found that emotion regulation and cognitive control were improved by activating the left DLPFC in MDD patients (30). It should be noted that the mean active oxy-Hb values of channels 1, 3, 11, 12 and 17 were not significantly increased than those at the baseline in both two groups in our study. Possible explanations for these findings may include the absence of long-term follow-up, which may indicate a need for additional therapy sessions, considering that most depressive episodes last for at least a few months. Besides, the SFBT mean integral value of oxy-Hb in T3 was negative in the study. Possible explanations may be motion artifacts and physiological factors such as blood pressure, heart rate, and respiratory rate can affect the oxy-Hb levels and lead to a negative integral value (31).

This study found that the number of words in the VFT task of the SFBT group was gradually significantly increased after the third intervention. However, the AC group exhibited a gradual decline in VFT task performance, which was opposite to the trend observed in the SFBT group. Thus, these findings suggested that a performance improvement in cognitive capacity accompanied with SFBT therapy. The neural signatures of anxiety and depressive symptoms within the prefrontal cortex (PFC) were found to be similar when using fNIRS, had less cortex activation during the tasks compared to healthy controls (HCs) particularly in the medial subregions, indicating a potential shared neural basis for these symptoms during late adolescence (32, 33). Zhang et al. found that MDD patients had considerably decreased PFC activation and were associated with reduced rise in oxy-Hb in prefrontal regions when undertaking cognitive tasks relative to controls (34). Tseng et al. showed that there was a negative correlation between state anxiety and the right lateralization index in the ventrolateral prefrontal cortex (VLPFC) in fNRIS during the period of n-back working memory task (35). However, the results about the clinical correlations between cognition and depression symptom severity were still remains controversial as several studies suggested that a correlation existed, while others did not (36, 37). The difference between these results may be related to the inconsistency in the patient characteristics between studies or the letters used and different time settings in the VFT (38).

### Limitations

There were several limitations to the current study. First, our study used non-randomized design but we tried to clearly report the study design, methods, and any limitations in details based on Transparent Reporting of Evaluations with Non-randomized Designs (TREND) statement. Employing a single-blind design, participants were kept unaware of the specific details of the psychological treatment they received, yet informed about the study’s general purpose and potential implications. All participants adhered to standardized procedures, conducted in a dedicated location separate from treatment sessions, to control for procedural variances.

Second, the relatively small percentage of patients included in the follow-up examination may have caused a bias. In fact, a comparison between the 53 patients analyzed in this study and the remaining 76 patients that were not included showed a significant difference in the sex ratio. Future research should aim to address this limitation by recruiting a more balanced sample or exploring the role of gender by regressing sex ratio or gender-sensitive statistical analyses, which would provide a more comprehensive understanding of the effects being studied across different genders.

Third, the study did not control the medications taken by participants, and thus, the potential influence of drugs on the findings cannot be ruled out. No clear evidence demonstrated effects of these drugs on the NIRS signals. In some brain regions, however, diazepam equivalent doses were related to NIRS signals in a cross-sectional analysis. In the future, it is therefore necessary to elucidate the influence of medication on NIRS signals in more detail. Furthermore, we should take into account pre-disease IQ, cognitive reserve, and the prevalence of cognitive impairment. In future research, we will further improve these shortcomings.

## Conclusions

Altogether, our results support that SFBT can improve depressive and anxiety symptoms as well as executive function of adolescent depression. Further studies will expand the sample size and have a longer follow-up to explore the mechanism of brain regulation.

## Data availability statement

The raw data supporting the conclusions of this article will be made available by the authors, without undue reservation.

## Ethics statement

The study was approved by the Institutional Review Board (IRB) of the Affiliated Hospital of Hangzhou Normal University (Hangzhou, China; 2022 (E2)-HS-076). The studies were conducted in accordance with the local legislation and institutional requirements. Written informed consent for participation in this study was provided by the participants’ legal guardians/next of kin.

## Author contributions

HC, MZ and ZY conceptualized the study and funding proposal, as well as drafted the manuscript. HL, LH and AM revised the manuscript and provided expert consultation on the design of the study. HC and MZ analyzed the data. All authors contributed to the article and approved the submitted version.
