# Corrigendum: Efficacy and executive function of solution-focused brief therapy on adolescent depression

**DOI:** 10.3389/fpsyt.2024.1417877

**Published:** 2024-04-26

**Authors:** Haisi Chen, Mengmeng Zhou, Li Han, Advaith Manoharasetty, Zhenghe Yu, Hong Luo

**Affiliations:** ^1^Affiliated Mental Health Center & Hangzhou Seventh People’s Hospital, Zhejiang University School of Medicine, Hangzhou, China; ^2^Internal Medicine Department, Hangzhou Linping District Maternal and Child Care Hospital, Hangzhou, China; ^3^Institute for International Education, Zhejiang Chinese Medical University, Hangzhou, China

**Keywords:** Solution-Focused Brief therapy, psychodynamic psychotherapy, adolescent, Major depressive disorder, Functional near-infrared spectroscopy

In the published article, there was an error in affiliation 1. Instead of “Affiliated Mental Health Center & Hangzhou Seventh People’s Hospital, Zhejiang University, Hangzhou, China”, it should be “Affiliated Mental Health Center & Hangzhou Seventh People’s Hospital, Zhejiang University School of Medicine, Hangzhou, China”.

The authors apologize for this error and state that this does not change the scientific conclusions of the article in any way. The original article has been updated.

